# *POFUT1* promotes colorectal cancer development through the activation of Notch1 signaling

**DOI:** 10.1038/s41419-018-1055-2

**Published:** 2018-09-24

**Authors:** Yuheng Du, Daojiang Li, Nanpeng Li, Chen Su, Chunxing Yang, Changwei Lin, Miao Chen, Runliu Wu, Xiaorong Li, Gui Hu

**Affiliations:** Department of Gastrointestinal Surgery, The Third Xiangya Hospital of Central South University, Changsha, Hunan Province China

## Abstract

Copy number variations (CNVs) are key drivers of colorectal cancer (CRC). Our previous studies revealed that protein O-fucosyltransferase 1 (*POFUT1*) overexpression is driven by CNVs during CRC development. The potential role and underlying mechanisms of *POFUT1* in CRC were not investigated. In this study, we analyzed the expression of *POFUT1* in CRC from cosmic and TCGA databases and confirmed that *POFUT1* is highly expressed in CRC. We used well characterized CRC cell lines, including SW620 and HCT116 to establish a model *POFUT1* knockdown cell line. Using these cells, we investigated the role of *POFUT1* in CRC. Our data revealed that silencing *POFUT1* in CRC cells inhibits cell proliferation, decreases cell invasion and migration, arrests cell cycle progression, and stimulates CRC cell apoptosis in vitro. We further demonstrate that *POFUT1* silencing dramatically suppresses CRC tumor growth and transplantation in vivo. We additionally reveal new mechanistic insights into the role of *POFUT1* during CRC, through demonstrating that *POFUT1* silencing inhibits Notch1 signaling. Taken together, our findings demonstrate that *POFUT1* is a tumor activating gene during CRC development, which positively regulates CRC tumor progression through activating Notch1.

## Introduction

Colorectal cancer (CRC) is a leading cause of cancer-related deaths in both economically developed and developing countries^1^. CRC remains one of the most important global malignancies and according to recent statistics, represents the fastest growing cancer in China^2^.

Numerous studies have explored CRC pathogenesis. It is known that CRC has a poor prognosis due to the proliferative, migratory and invasive capabilities of CRC tumors^3,4^. CRC cells can be influenced by various intrinsic and extrinsic factors, including hormones, cytokines, and oncogenes^5–8^. Our previous studies identified copy number variations (CNVs) in CRC that led to genes located on chromosome 20q11 being upregulated^9^. One of these genes was identified as protein O-fucosyltransferases 1(*POFUT1*). The contribution of *POFUT1* to CRC development was not investigated.

Glycosylation regulates many physiological and pathological processes, including inflammation, tumor progression, and embryo implantation^10–12^. Fucosylation is an important type of post-translational modification of proteins contributing to N-glycan and O-fucosylglycan elaboration. Fucosylation is the process of adding fucose units onto a molecule through a process catalyzed by fucosyltransferases (FUTs) and protein O-fucosyltransferases (POFUTs)^13^, including *POFUT1*. Recent studies reveal that *POFUT1* is closely associated with Dowling-Degos disease^14^, and certain solid cancers including breast cancer and liver cancer^15–17^. The major cellular function of *POFUT1* is the activation of Notch1 signaling, as *POFUT1* promotes binding of Notch1 ligands, increasing receptor activation, cleavage and nuclear translocation of notch intracellular domain 1(NICD1)^18–20^.

Notch signaling is an essential cell–cell communication pathway conserved in all metazoan organisms^18^. Notch signaling has emerged as a critical signaling pathway during carcinogenesis due to its ability to regulate multiple proliferative and metastatic processes^7,21–24^. In mammals, four Notch receptors (Notch1–4)^25^ and five ligands (Jag1, Jag2, Dll1, Dll3, and Dll4) mediate these signaling events. Binding of Notch ligand to its cognate receptor triggers cleavage and release of NICD^26^, which shuttles to the nucleus to transcriptionally activate target genes, including *H*es1, Hey1^27–29^ and other responsive genes, including *c-*Myc^21^. Different Notch signaling pathways contribute to a myriad of cell signaling processes that in turn, regulate a range of cellular functions. The Notch1 signaling pathway has been shown to be overactive in CRC tumors^21,30,31^. Notch 1 promotes CRC invasiveness through activating several pro-oncogenic factors, including *CD44, CCND1*, and *Bcl-2*^21,32–35^. The contribution of *POFUT1* overexpression to Notch1 activity in CRC, has not been defined.

In this study, we investigated the role of *POFUT1* during CRC development and its contribution to tumor growth and metastasis. We investigate the downstream effects of aberrant POFUTI expression and assess the effects of *POFUT1* on Notch1 signaling in CRC.

## Results

### *POFUT1* is highly expressed in human CRC

**Fig. 1:**
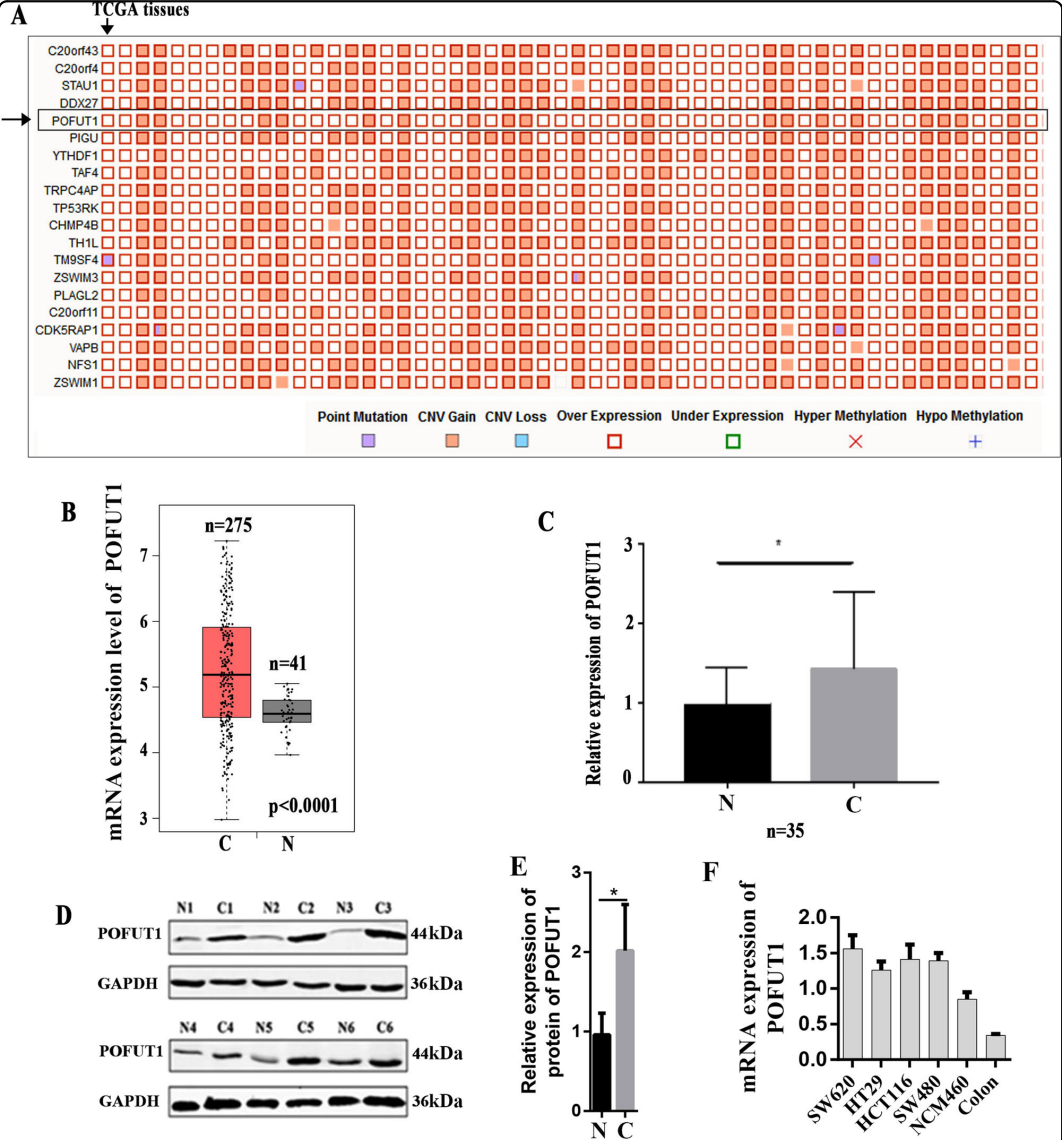
*POFUT1* is overexpressed in colorectal cancer tissues. **a** The top 20 overexpressed genes in colorectal cancer tissues from Cosmic database (https://cancer.sanger.ac.uk/cosmic/). A “Mutation Matrix” plot between genes and samples for colorectal tissue which contains top 20 ranked genes (rows) and TCGA samples (columns) with each box representing a Gene-Sample combination. POFUT1 genes ranked 5in colorectal cancer tissues (Only some TCGA samples are listed here, more details can be found in website). **b** The mRNA expression of *POFUT1* in colorectal cancer tissues from TCGA colorectal cancer tissues, this figure generated by GEPIA (http://gepia.cancer-pku.cn/index.html), which is a newly developed interactive web server for analyzing the RNA sequencing expression data of 9736 tumors and 8587 normal samples from the TCGA and the GTEx projects, using a standard processing pipeline. (The number of cancer tissue (C) is 275; the number of normal tissue (N) is 41). **c** The mRNA expression of *POFUT1* in our 35 pairs of human CRC tissues (C) and adjacent non-tumor tissues (N). *p < 0.05. **d** The protein expression of *POFUT1* in six representative pairs of primary CRC (N) and adjacent non-tumor tissues (N). **e** Quantification of **d**. *p < 0.05. **f** The mRNA expression of *POFUT1* in four colorectal cells lines (SW480, SW620, HCT116, and HT29); a normal human colon epithelial cell lines (NCM460) and human normal colon tissues

To investigate the roles of *POFUT1* during CRC development, first, we try to investigate the expression of *POFUT1* in CRC tissues. The Catalogue Of Somatic Mutations In Cancer (COSMIC)^36^, the world’s largest and most comprehensive resource for exploring the impact of somatic mutations in human cancer, was used to analyze the expression of *POFUT1* in colorectal cancer tissues, the result found that overexpression of *POFUT1* occurred in 53.44% (326/610) CRC tissues. Additionally, COSMIC have identified that *POFUT1* overexpression mainly occurred in CRC tissues and ranked first in all human tumor tissues, and *POFUT1* belonged to the top 20 genes (ranked 5) with extremely high frequency of overexpression in CRC (Fig. 1a). Then, online database GEPIA^37^, which is a newly developed interactive web server for analyzing the RNA sequencing expression data of tumors and normal samples from the TCGA projects, was used to assess the expression of *POFTU1* in human CRC tissue and non-tumor tissue, the result found that the expression level of *POFUT1* is significantly higher in colorectal cancer tissues than normal tissues (Fig. 1b). To further confirms this conclusion, 35 matched pairs of human CRC tissue, and adjacent non-tumor tissue was selected for quantitative polymerase chain reaction (q-PCR) and western blot analysis, the result demonstrated that *POFUT1* is significantly upregulated in human CRC tissue (Fig. 1c–e), suggesting its association with CRC progression. Additionally, we determined the mRNA expression of *POFUT1* in 4 colorectal cells lines (SW480, SW620, HCT116, and HT29), a normal human colon epithelial cell lines (NCM460) and human normal colon tissues. It was found that *POFUT1* is elevated in colon cancer cells compared to normal colon epithelial cell line and normal colon tissues, which further implicating its contribution to CRC pathogenesis (Fig. 1f).

### *POFUT1* silencing reduces CRC cell proliferation in vitro

**Fig. 2:**
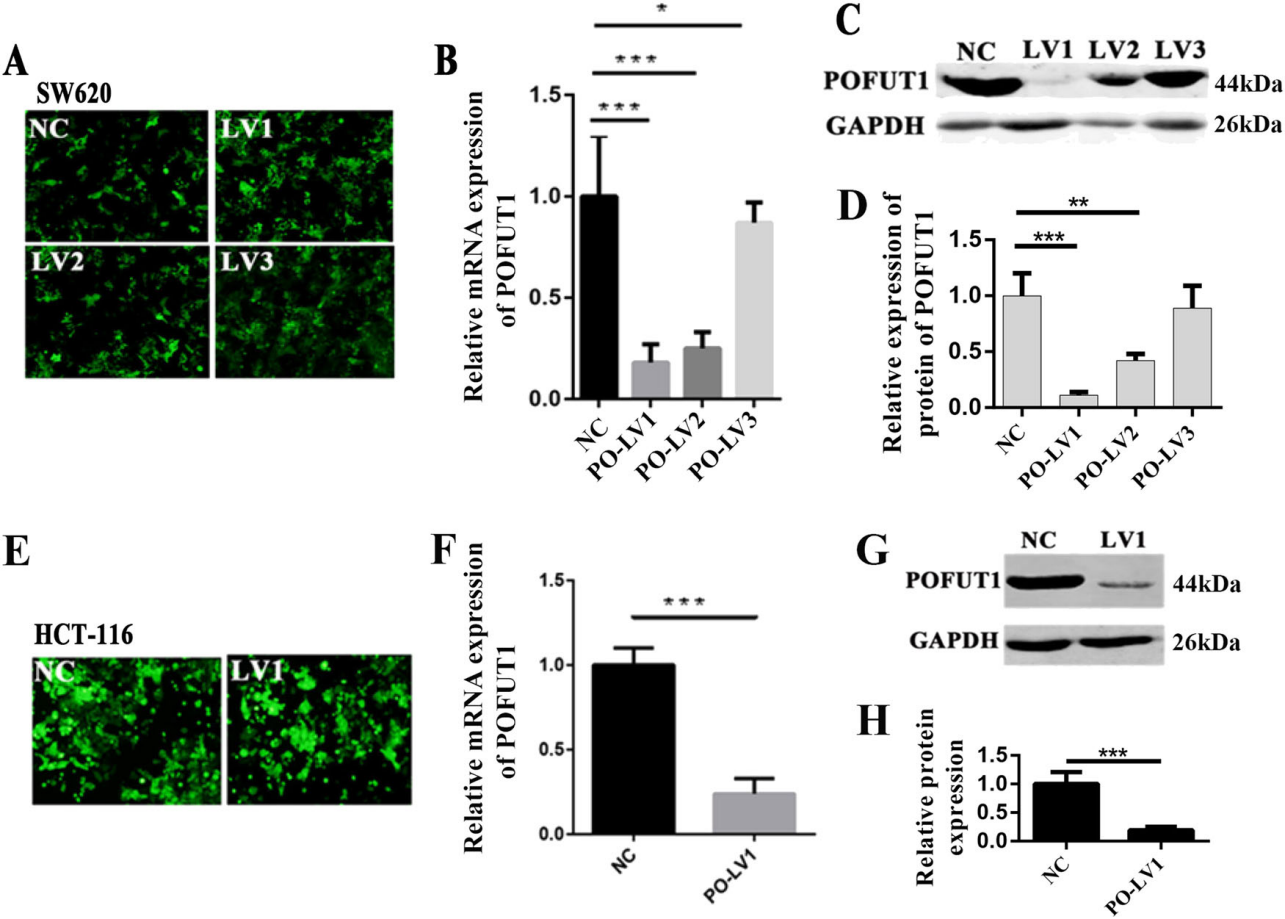
The model of knockdown cell lines. **a** GFP in lentivirus transfected SW620 cell line. **b** Relative mRNA levels of *POFUT1* were measured by real-time q-PCR assays after transfection of negative control (NC) or three different shRNAs (shPOFUT1*–*1:PO-LV1, shPOFUT1*–*2:PO-LV2, and shPOFUT1*–*3: PO-LV3) in SW620 cells. *p < 0.05, ***p < 0.01. **c** Protein levels of *POFUT1* were measured by western blot assays after transfection of negative control (NC) or three different shRNAs (shPOFUT1*–*1:PO-LV1, shPOFUT1*–*2:PO-LV2, and shPOFUT1*–*3: PO-LV3) in SW620 cells. **d** Quantification of **c**. *p < 0.05, ***p < 0.01. **e** GFP in lentivirus transfected HCT116 cell line. **f** Relative mRNA levels of *POFUT1* were measured by real-time q-PCR assays after transfection of negative control (NC) or shPOFUT1 (PO-LV1) in HCT116 cells. *p < 0.05, ***p < 0.01. **g** Protein levels of *POFUT1* were measured by western blot assays after transfection of negative control (NC) or shPOFUT1 (PO-LV1) in HCT116 cells. **h** Quantification of **c**. ***p < 0.01, **p < 0.03

We next investigated the cellular effects of *POFUT1* silencing in SW620 and HCT116 CRC cell lines. *POFUT1* was first silenced through transfection of lentiviral *POFUT1* shRNAs into SW620 cells. GFP was co-transfected in these cells to confirm transfection efficiency (Fig. 2a). *POFUT1* expression was examined in both *POFUT1* knockdown cells (SW620-PO-Lv1, SW620-PO-Lv2, and SW620-PO-Lv3) and scrambled shRNA controls(SW620-NC). q-PCR and western blot analysis showed that the expression of *POFUT1* was dramatically decreased in knockdown cells, with Lv1 displaying the most efficient levels of *POFUT1* silencing (SW620-PO-Lv1, *p* < 0.01, Fig. 2b–d). Given these results, we transfected Lv-1 into HCT116 cells and assayed *POFUT1* expression (Fig. 2e). We again observed a significant reduction in *POFUT1* mRNA and protein expression in HCT116 PO-Lv1 cells compared to HCT116-NC controls, confirm comparable silencing to that observed in SW620 cells (*p* < 0.01, Fig. 2f, g). These stably transfected PO-Lv1 cells were used to investigate the cellular roles of *POFUT1* in subsequent cell-based experiments.

**Fig. 3:**
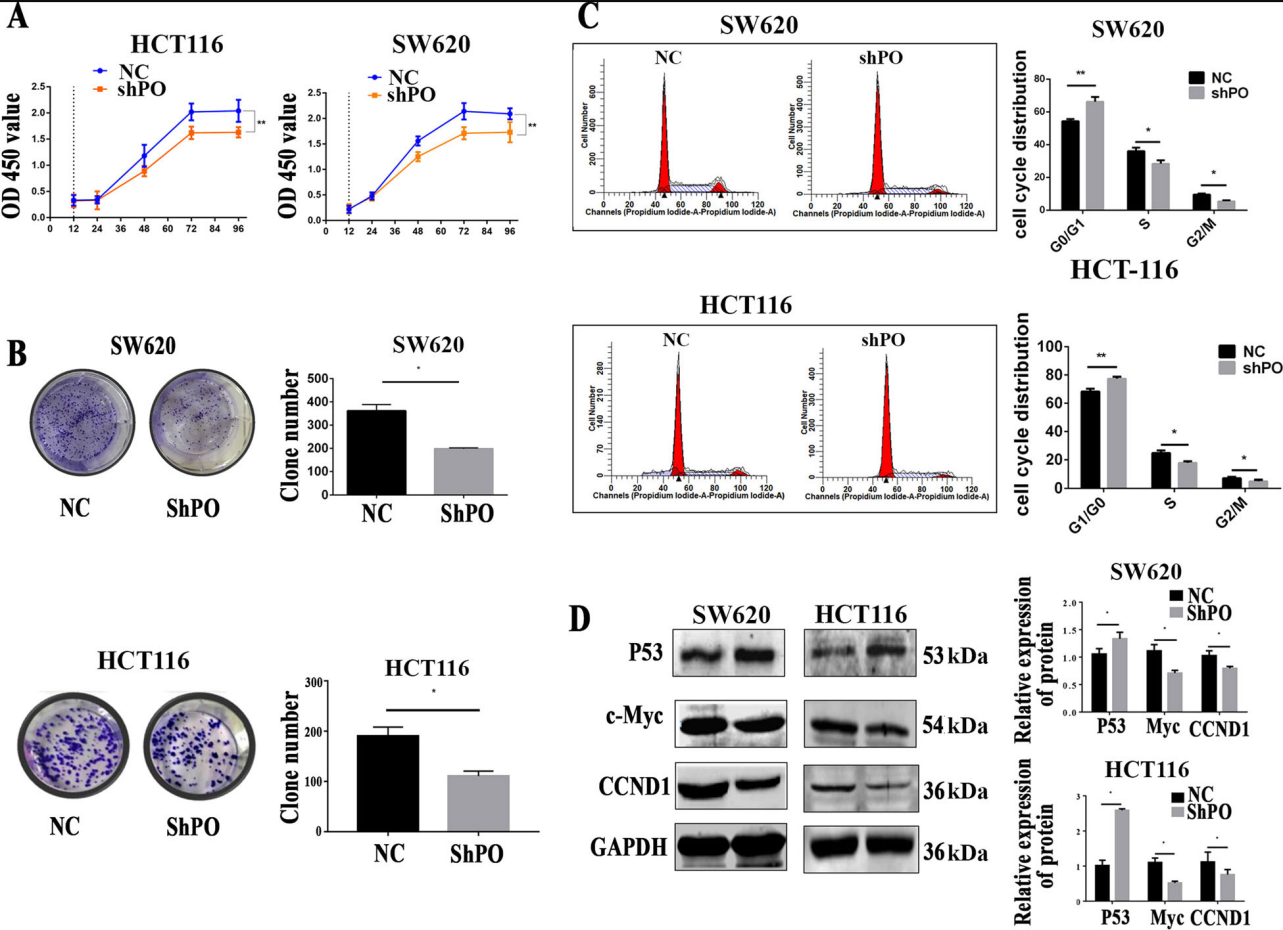
Knockdown of *POFUT1* significantly inhibited CRC proliferation in vitro. **a** Compared negative control group (NC). Knockdown of shPOFUT1 (shPO) significantly reduced CRC cell proliferation ability by CCK8 assay in SW620 and HCT116 cell lines. **p < 0.03. **b** Compared negative control group (NC). Knockdown of shPOFUT1 (shPO) decreased CRC cell colony numbers in SW620 and HCT116 cell lines. Quantification (right panel), *p < 0.05. **c** *POFUT1* arrested the progression of the cell cycle in SW620 and HCT116 cell lines. Quantification (right panel), these data were detected by a flow cytometry. *p < 0.05, **p < 0.03. **d** Western blot assay revealed the level of p53 increased and the level of protein CCND1 and c-Myc decreased in the *POFUT1* group compared with the NC group. *p < 0.05

Then CCK-8 (Fig. 3a) and colony formation assays (Fig. 3b) were performed, *POFUT1* knockdown significantly inhibited cell growth relative to NC controls (*p* < 0.05). Cell cycle analysis also revealed an decreased 66.22% ± 1.67% of cells in the G0/G1 phase of the cell cycle, a decreased 28.36% ± 1.25% of cells in S phase and a decreased 5.42% ± 0.39% of cells in G2/M phase in shPOFUT1 groups compared with 54.32% ± 0.80% of cells in the G0/G1 phase, 36.10% ± 1.18% of cells in S phase and 9.58% ± 0.37% in the G2/M phase in NC groups. Similarly in HCT116 groups. Cell cycle analysis also revealed an increased 77.28% ± 0.89% of cells in the G0/G1 phase of the cell cycle, a decreased 17.80% ± 1.31% of cells in S phase and a decreased 4.92 ± 0.51% of cells in G2/M phase, compared with 68.23% ± 1.14% in G0/G1 phase, 24.79% ± 1.19% in S phase and 6.98% ± 0.61% in G2/M phase in NC groups. This suggested that cell cycle progression is arrested following *POFUT1* silencing (Fig. 3c, *p* < 0.05).

We further assessed the expression of cell proliferation-related proteins in SW620-PO-Lv1 and HCT116-PO-Lv1 cells (Fig. 3d). As *p53* played an important role of inhibiting tumor growth and proliferation in CRC^38^, *c-Myc* were overexpressed in CRC and linked with CRC proliferation intimately^39^, *CCND1* were closely connected with cell cycle and tumor proliferation^40^. We carried out the experiment to examine the expression levels of *p53*, *c-Myc*, and *CCND1* in cell lines. *POFUT1* knockdown was found to significantly decrease c-Myc and CCND1 protein expression, and increase p53 protein expression compared to NC controls (*p* < 0.05). Taken together, we therefore conclude that *POFUT1* silencing leads to cell cycle arrest in model CRC cell lines.

### *POFUT1* silencing reduces CRC cell migration and invasion

**Fig. 4:**
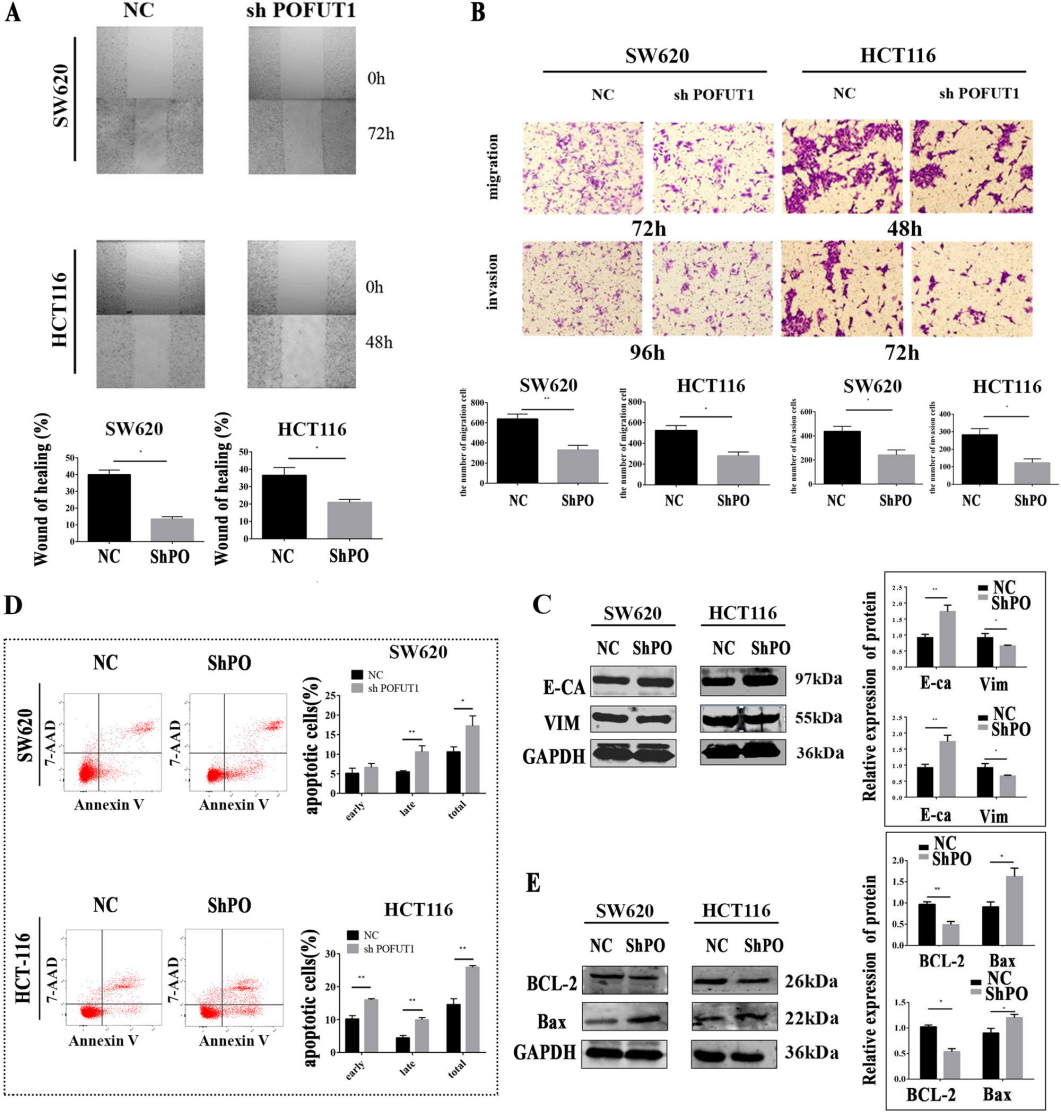
Downregulation of *POFUT1* inhibits CRC cell migration and invasion and promotes CRC cell apoptosis in vitro. **a** The wound-healing assay showed that the area of co-colonization in the shPOFUT1 group was lower than the NC group. Quantification (bottom panel), *p < 0.05, magnification: ×40 **a**. **b** The migration and invasion ability of SW620 and HCT116 cells were significantly inhibited in the shPOFUT1 group relative to the NC group, which was evaluated using a transwell assay. Quantification (bottom panel), *p < 0.05, **p < 0.03, magnification: ×200 **b**, **c**. **c** A western blot assay revealed the level of E-cadherin increased and the level of protein Vimentin decreased in the shPOFUT1 group compared with the NC group. Quantification (right panel), *p < 0.05, **p < 0.03. **d** A flow cytometry assay indicated that the percent of total apoptotic cells significantly increased in the shPOFUT1 group. Quantification (right panel), *p < 0.05, **p < 0.03. **e** A western blot assay revealed the level of Bax increased and the level of protein bcl-2 decreased in the shPOFUT1 group compared with the NC group. Quantification (right panel), *p < 0.05, **p < 0.03

Through wound-healing assays, we evaluated the biological effects of *POFUT1* on CRC cell migratory behavior; a vital change of CRC metastasis and tumor progression. *POFUT1* silenced cells displayed notable decreases in re-colonization into the wound region after 72 h in SW620-PO-Lv1 cells or 48 h for HCT116-PO-Lv1 cells (*p* < 0.05, Fig. 4a). In transwell assays, *POFUT1* silencing reduced the ability of CRC cells to migrate in comparison to NC groups after 72 h for SW620-PO-Lv1 cells (331 ± 45.82 vs SW620-NC:638.8 ± 48.39 per field) and 48 h in HCT116-PO-Lv1 cells (281 ± 36.3 vs HCT116-NC:524 ± 48.5 per field). A reduction in transwell invasion was observed up to 96 h in SW620 cell lines (SW620-PO-Lv1:242.3 ± 41.31 vs SW620-NC:438.3 ± 41.29 per field), and 72 h in HCT116 cell lines (HCT116-PO-Lv1:282.3 ± 35.34 vs HCT116-NC:123 ± 22.46 per field), Fig. 4b (*p* < 0.05). We further examined the expression of migration and invasion-related proteins following *POFUT1* silencing in SW620 and HCT116 cells (Fig. 4c). Western blot revealed that *POFUT1* silencing significantly deceased the expression of *Vimentin*, but increased E-cadherin expression relative to NC controls. These data suggest that epithelial-mesenchymal transition (EMT) of CRC cells is impaired following *POFUT1* silencing (*p* < 0.05).

### *POFUT1* silencing promotes CRC cell apoptosis

CRC cell apoptosis was investigated by flow cytometry through V-APC and 7-AAD staining of SW620-PO-Pv1 and HCT116-PO-Lv1 cells. *POFUT1* silenced SW620-cells (17.23% ± 2.57%) and HCT116-cells (25.91% ± 0.51%) showed higher percentages of cells undergoing apoptosis, compared to NC controls (10.56% ± 1.33 in SW620 cells, 14.54% ± 1.78% in HCT116 cells), (Fig. 4d, *p* < 0.05). When we investigated the expression of apoptotic proteins in these cell lines, densitometry analysis of western blots revealed that *POFUT1* silencing significantly decreased the expression of *Bcl-2*, and increased *Bax* levels, compared to NC cells (Fig. 4e, *p* < 0.05).

### *POFUT1* silencing reduces CRC tumor growth and migration in vivo

**Fig. 5:**
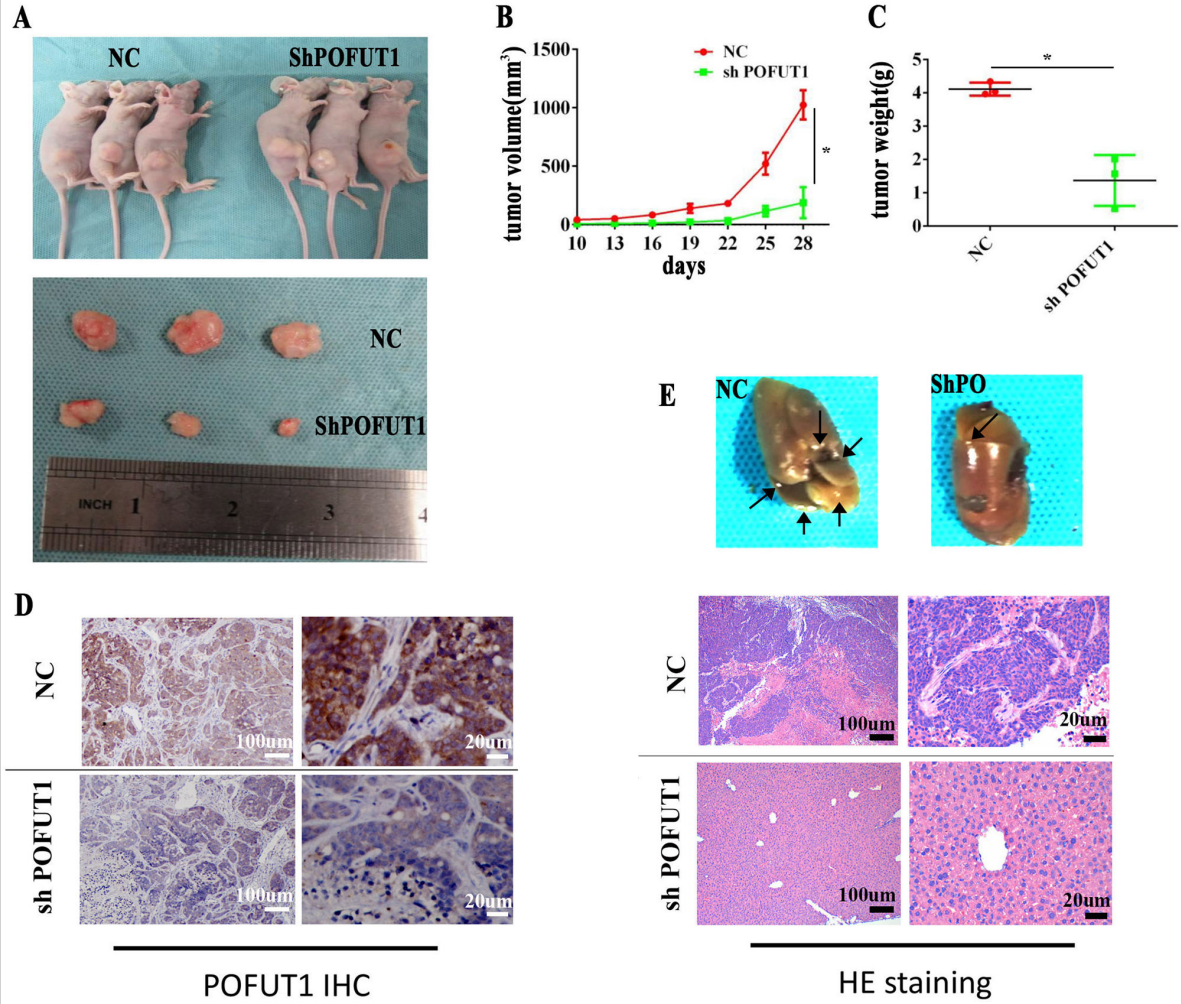
Knockdown of *POFUT1* inhibited tumor progress in vivo. **a** Compared with the NC group, *POFUT1* significantly decreased tumor size **b** and tumor weight **c** at the end of the experiment. Right panel showed representative images of SW620 xenograft tumors. Knockdown of *POFUT1* in tumors from SW620-shPOFUT1 was confirmed by immunohistochemistry (IHC). **d** Magnification: ×10 (left, scale: 100 µm), ×40 (right, scale: 20 µm). *p < 0.05. **e** The metastatic lession of tumor in liver was less in shPOFUT1 groups evaluated by tail vein injection and H&E staining. Magnification: ×10 (left, scale: 100 µm), ×40 (right, scale:20 µm). *p < 0.05

We have thus far revealed that *POFUT1* silencing inhibits CRC cell proliferation, migration and invasion in vitro. We next aimed to confirm our findings using in vivo models of CRC. To achieve this, SW620-PO-Lv1 cells and SW620-NC cells were used to assess CRC tumor formation in nude mice. The data showed *POFUT1* promotes CRC tumor growth in vivo (Fig. 5a) as tumor size (188.146 ± 78.359 mm^3^) and mice weight (1.365 ± 0.765) were significantly lower in SW620-PO-Lv1 cells compared to SW620-NC controls (size = 1024.508 ± 124.930 mm^3^, weight = 4.109 g ± 0.1986 g), (Fig. 5b, c). *POFUT1* silencing in SW620-PO-Lv1 tumors was confirmed by IHC assessment (Fig. 5d). We next assessed the transplantation capabilities of *POFUT1* silenced tumors via tail vein injections in nude mice (Fig. 5e). At the end of the experiment, nude mice were killed and their livers removed for pathological analysis. Hematoxylin and eosin (H&E) staining results revealed significantly fewer liver metastasis in *POFUT1* silenced cells, compared to NC controls.

### *POFUT1* knockdown downregulates Notch 1 signaling

**Fig. 6:**
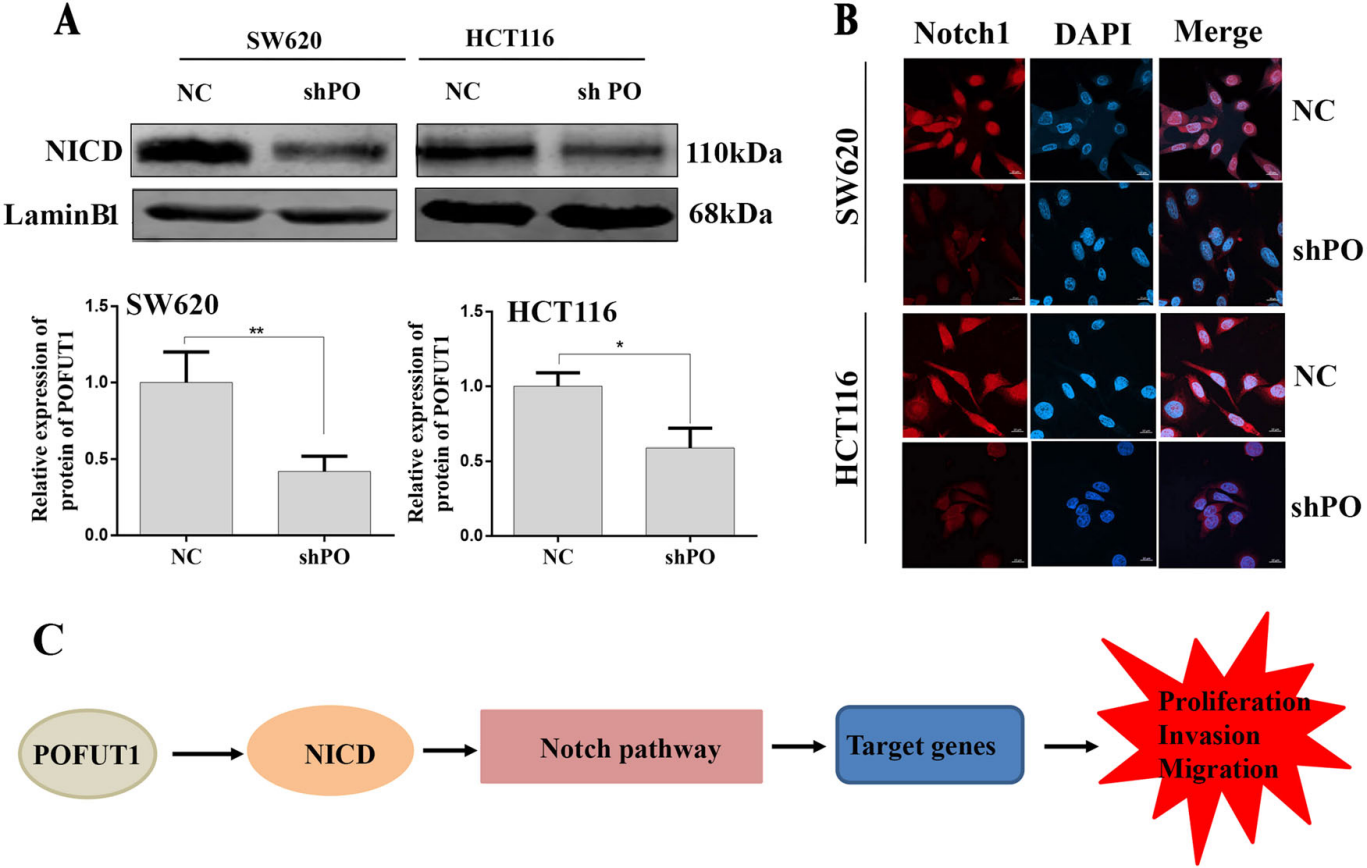
*POFUT1* knockdown downregulates Notch1 signaling pathway. **a** The western blot assay indicated that the nuclear protein level of NICD in the shPOFUT1 group were significantly lower than that in the NC group. *p < 0.05, **p < 0.03. **b** The immunofluorescence assay showed the expression of the proteins NICD in the shPOFUT1 group were siginicicantly lower than in the NC group. Magnification, ×60. **c** Overexpression of *POFUT1* led to the NICD1 increase in nucleus, Notch1 signaling pathway highly activated and target genes were overexpressed, finally promoted CRC proliferation, invasion and migration

We speculated that the effects of *POFUT1* silencing on CRC proliferation, migration and invasion may be intimately linked with the ability of *POFUT1* to activate Notch1 signaling in CRC cells. Western blot assays were employed to detect changes of NICD in cell nucleus. Notably, *POFUT1* silenced cells displayed significantly decreased NICD cleavage levels in SW620 and HCT116 nucleus (Fig. 6a). Furthermore, immunofluorescent analysis of the cellular localization of NICD revealed reduced nuclear translocation in SW620 and HCT116 cell lines knockdown for POFUT1 (Fig. 6b). Collectively, these data suggest that in CRC, overexpression of *POFUT1*, highly activates Notch1 signaling to promote CRC cell proliferation, invasion, and migration in vivo (Fig. 6c).

## Discussion

Overwhelming evidences have shown that lots of molecules, which are expressed in CRC, have crucial roles in CRC tumor development^5,41^. Abnormal expression of these signaling molecules leads to the downstream activation of pro-oncogenic signaling pathways that ultimately lead to aberrant proliferation, invasion, and migration of tumor cells. Recent studies indicate the role of glycosyltransferases in tumor cell progression. Examples include *FUT5* and *FUT6* that mediate CRC cell proliferation, invasion, and migration^42^. *POFUT1* is a member of the glycosyltransferase O-Fuc family that adds fucose through glycosidic linkages to serine or threonine residues of many cellular proteins. Accordingly, its dysregulation is intimately linked to human disease, particularly Dowling-Degos disease; a rare genetic disease that results in skin hyperpigmentation^18^. During cancer development, though a role has been implicated in solid cancers, the contribution of *POFUT1* to CRC was less well understood. Here, we reveal a number of oncogenic phenotypes of CRC cell lines that are dependent on *POFUT1* expression. *POFUT1* silenced CRC cell lines display reduced proliferation, (assessed using CCK-8 and colony formation assays), cell cycle arrest, reduced invasiveness (assessed using wound-healing and Transwell assays) and higher levels of apoptosis. We confirmed the importance of *POFUT1* to promote CRC tumor formation in vivo using mouse models of CRC development. The combination of these data implicate *POFUT1* as a crucial CRC oncogene, the overexpression of which is intrinsically linked to CRC tumor development.

Tumor cell proliferation and metastasis are key steps during CRC malignancy, both of which are regulated to intracellular kinase signaling^43^, including wnt-βcatenin signaling, Notch signaling, p53 expression and PI3K/Akt signaling^21,43–48^. *POFUT1* is known to regulate O-fucosylation of specific members of these signaling cascades, with its role during *Notch1* signaling well-defined^49,50^, O-fucosylation on Notch receptor epidermal growth factor (EGF)-like repeats is catalyzed by the protein *O*-fucosyltransferase 1 (*POFUT1*) and primarily controls Notch interaction with its ligands^26^. In this article, our study showed that gene protein *O*-fucosyltransferase 1 (*POFUT1*), which encodes a glycosyltransferase required for Notch1 signaling^25^, has increased expression in CRC. Notch receptors undergo γ-secretase-mediated proteolytic cleavage, which releases the Notch intracellular domain (NICD) from the membrane. NICD subsequently translocates to the nucleus and forms a complex with the DNA-binding factor RBP-Jκ(CSL) which switches-on the transcription of target genes^20^. Besides in some solid cancers, *POFUT1* activate Notch1 signaling pathway in other pathological progress, such as to explore the effect of O-fucosylation in myogenesis, Audrey Der Vartanian and her colleagues study a murine myoblastic C2C12 cell line and inhibited Pofut1 expression by short hairpinRNA (shRNA) during the time course of differentiation, the result found that knockdown of Pofut1 affected the signaling pathway activation by a reduction of the amount of cleaved Notch intracellular domain and a decrease in downstream Notch target gene expression^26^. Similarly, Zygmunt et al. found that *POFUT1* deletion in skeletal myofibers can reduce Notch signaling in young adult muscles, but this effect was lost with age^25^. In this study, we confirmed the ability of *POFUT1* to regulate *Notch1* signaling in CRC, as *POFUT1* silencing downregulated NICD translocation to the nucleus following Notch1 activation. This provided mechanistic insights into the pro-oncogenic effects of *POFUT1* upregulation in CRC tumors.

This study had some limitations. First, the expression of *POFUT1* was examined in 35 paired tissues using real-time q-PCR and western blot analysis. Larger sample sizes are required to confirm its upregulation in CRC tumor tissue. Secondly, the pro-oncogenic mechanisms of *POFUT1* expression during CRC development could be investigated in more detail. Further experiments investigating the effects of pharmacological O-fucosylation inhibition on CRC tumors would validate *POFUT1* as a drug target. In addition, oncogenic changes to “healthy” colorectal tissue following exogenous overexpression of *POFUT1* would reveal this protein as a direct driver of CRC. In these tissues, the ability of exogenous *POFUT1* to over-stimulate Notch 1 signaling would further validate this as the mechanism responsible for the pro-oncogenic functions of *POFUT1*.

In conclusion, to our knowledge, this is the first study to report the role of *POFUT1* during CRC proliferation, invasion, and migration. Our data demonstrate that the pro-oncogenic effects of *POFUT1* are mediated through Notch1 signaling. The identification of *POFUT1* expression driven by CNVs provides new information for the both the diagnosis and treatment of CRC. The potential of *POFUT1* inhibition as a novel drug target to impede CRC tumor progression, now warrants further investigation.

## Materials and methods

### Tissue samples

CRC specimens and adjacent tissues were obtained from the Third Affiliated Hospital of Central South University (Changsha China) from January to April, 2017. All the samples had a clear histologic diagnosis of CRC assessed by experienced pathologists. All samples were obtained from patients who had not received chemotherapy or radiotherapy prior to the operation. The protocols for human studies were in accordance with the Institute Research Medical Ethics Committee of Central South University, and each patient signed individual consent forms.

### Cell culture

Human CRC cells SW620, HCT116, SW480, HT29, and normal colonic epithelia cells (NCM460) were purchased from Boster Company (Boster, Wuhan, People’s Republic of China). SW620 and SW480 were grown in L15 medium (KeyGEN BioTECH, Nanjing, China) supplemented with 10% FBS (Biological Industries, Israel). HCT116, HT29, and NCM460 cells were grown in McCoy′s5A medium (KeyGEN BioTECH, Nanjing, China) supplemented with 10% FBS (Biological Industries, Israel). All cells were incubated at 37 °C with 5% CO_2_.

### Quantitative real-time PCR

Cells and tissues were treated with Trizol reagent (Invitrogen, Carlsbad, CA, USA) for RNA extraction. cDNA synthesis and real-time quantitative PCR (q-PCR) were performed using the TOYOBO RT kit and KOD SYBR® q-PCR kit (TOYOBO Co., Ltd, Osaka, Japan). Primers used in the study were as follows: *POFUT1*: 5′-AACCAGGCCGATCACTTCTTG-3′ (Forward), and 5′-GTTGGTGAAAGGAGGCTTGTG-3′ (Reverse). *GAPDH*: 5′-GCACCGTCAAGGCTGAGAAC-3′ (Forward), and 5′-TGGTGAAGACGCCAGTGGA-3′ (Reverse). Reactions were performed according to the manufacturer’s protocols using the Roche LightCycler 480 thermal cycler (Roche, Switzerland).

### Western blot analysis

Cells were lysed in lysis buffer (containing) and incubated for 30 min on ice. Lysates were clarified by centrifugation at 12,000 rpm for 10 min at 4 °C and supernatants were collected. For the nuclear protein, we chose the Nuclear and Cytoplasmic Protein Extraction Kit (KGP150, KeyGEN BioTECH, Nanjing, China). According to manual, briefly, cells were lysed in 450 μl buffer A and 50 μl buffer B, and incubated for 30 min on ice, after washing the precipitation by PBS, mixed the precipitation with 100 μL buffer C. Lysates were clarified by centrifugation at 12,000 rpm for 10 min at 4 °C. and supernatants were collected. Protein concentrations were determined using coomassie protein assay reagent using bovine serum albumin (BSA) as a standard. Approximately 50 μg of protein was separated by 10% SDS–PAGE (Well Biological Co., Ltd, Changsha, China), transferred to PVDF membranes (Invitrogen). Membranes were blocked in non-fat milk in PBST for 2 h at 37 °C and incubated overnight at 4 °C with the appropriate primary antibodies. Membranes were washed three times in TBST for 10 min at room temperature, and labeled with HRP-conjugated goat anti-rabbit IgG or anti-mouse IgG secondary antibodies for 120 min at room temperature. Membranes were washed a further three times (10 min per wash) in PBST, and bound antibodies were detected using HRP substrate. All samples were run in triplicate. Antibodies used in the study included: anti-POFUT1 (Proteintech, 14929–1-AP) 1:1000 dilution; anti-GAPDH (Abcam, ab181602) 1:2000 dilution; anti-c-Myc (Proteintech) 1:1000 dilution: anti-CCND1 (Proteintech) 1:1000 dilution; anti-Vimentin (CST) 1:1000 dilution; anti-E-cadherin (CST) 1:1000 dilution; anti-Bcl2 (CST) 1:1000 dilution, anti-Bax (CST) 1:1000 dilution; anti-p53 (CST) 1:1000 dilution, anti-lamin B1 (Santa Cruz Biotechnology, Dallas, TX) 1:1000 dilution; anti-Cleaved Notch 1 (Val1744) (D3B8) Rabbit mAb #4147, CST, this antibody detects endogenous levels of the Notch1 intracellular domain, but it does not recognize full-length Notch1 or Notch1 cleaved at other positions 1:1000 dilution.

### ShRNA lentiviral vectors and cell transfection

Lentivirus packaging and transduction supplied by the Shanghai GenePharma Co., Ltd. Three short hairpin RNAs targeting human POFUT1 (shPOFUT1–1: 5′-GGCATTTCCTTCAGTGCTTCC-3′, shPOFUT1–2: 5′-GGCCACTACAGAAGTACATGG-3′, and shPOFUT1–3: 5′-GCCCTATGTGGGCATTCATCT-3′) and a non-targeting RNA sequence (5′-TTCTCCGAACGTGTCACGT-3′) serving as a negative control were cloned into the vector majorly synthetized by Genepharma, Shanghai, China. Virus packaging was performed in HEK293T cells. To create lentivirus-transduced lines, cells were infected with shRNA lentiviral vectors and Polybrene (4 μg/ml), and stable cell lines were selected with treatment of 4 µg/ml puromycin after 48 or 72 h transfection. The efficiency in different cells was determined by qRT–PCR and WB.

### Cell colony formation

For colony formation assays, ~800 cells were seeded for 48 h and plated onto 6-well plates, which were cultured for 14 days. Resulting colonies were fixed in methyl alcohol, and stained with 5% crystal violet. The number of colonies per well was quantified using Image J 1.48v (National Institutes of Health, USA).

### Cell counting kit-8 assay

Cell proliferation was assessed using the CCK-8 kit (Dojindo Laboratories Co. Ltd, Kumamoto, Japan). Cells (4 × 10^3^ cells/well) were serum starved overnight and incubated in 96-well plates with 100 μl of normal culture medium at 37 °C, for the indicated time points. Ten microliters of CCK-8 solution was added to each well for 3 h at 37 °C. The absorbance values of each well (A450) were detected using an EnVision microplate reader (PerkinElmer). All experiments were performed in triplicate.

### Cell migration and invasion transwell assay

Cells were plated into the upper chambers of transwell (8 μm, 24-well insert; Corning, Lowell, MA, USA) at a density of 1 × 10^5^ per well. Chambers contained serum-free medium with or without Matrigel matrix (BD Biosciences) for cell migration and invasion assays, which were inserted into 24-well plates containing 20% FBS. After 36 h incubation, non-migrating and non-invasive cells in the upper chamber were removed with a cotton swab. Migrated or invasive cells that had adhered to the lower surface of chambers were fixed with methanol for 20 min and stained with 5% crystal violet 15 min. Cells were counted from eight random fields under a microscope.

### Cell cycle analysis

Cells were plated into 6-well plates at a density of 3 × 10^5^ per well for 24 h. Cells were fixed in 1% paraformaldehyde and stored in 70% ethanol at −20 °C. Cells were stained with 0.5 ml PI/RNase (BD Biosciences) staining buffer for 15 minutes at room temperature and analyzed by flow cytometry.

### Apoptosis assays using flow cytometry

Apoptosis flow cytometry assays were performed using APC Annexin V (BD BIOSciences, USA) and 7-AAD(BD Biosciences, USA). Cells were washed twice in cold PBS and resuspended in 1X binding buffer (BD Biosciences, USA) at a density of 1 × 10^6^ cells/ml. Cell suspensions (2.5 × 10^5^ cells) were added to 1.5 ml Eppendorf tubes to which 5 μl of APC Annexin V and 7-AAD were added and gently vortexed. Cells were incubated for 15 min at room temperature in the dark. Binding buffer (400 μl) was added to each tube and cells were analyzed by flow cytometry.

### Tumor formation and transplantation

Animal studies were approved by the Medical Ethics Committee to observe the formation and metastasis of CRC in vivo. SW620 cell lines were employed for tumor formation and transplantation assays. Each 6-week-old nude mouse was injected with 5 × 10^7^ cells for tumor formation models and 1 × 10^7^ cells were injected into the tail vein to establish tumor transplantation models. After 7 days, tumor sizes of the mice were recorded every 3 days. After 4 weeks, all subcutaneous injected nude mice were killed, we recorded the tumor sizes and weights. Tumor sizes were evaluated *V* = *π* × *L* × *W*^2^/6 (L: long diameter, W: wide) and assessed immunohistochemically. After 8 weeks, tail vein injected nude mice were killed, and the livers of the mice used for HE staining.

### Immunohistochemistry and immunofluorescence

Tissue slides were deparaffinized and rehydrated. Endogenous peroxidase activity was blocked with 0.3% hydrogen peroxide for 20 min. Slides were blocked in 10% BSA for 10 min, and incubated with anti-human *POFUT1* antibodies (1:100; Proteintech) at 4 °C for 12 h. Slides were incubated in biotinylated secondary antibodies at 37 °C for 15 min, and reacted with streptavidin-peroxidase conjugate at 37 °C for 10 min. 3,3′-diaminobenzidine was then added as a chromogen substrate. Negative controls were obtained by replacing primary antibodies with PBS. Images were captured using an inverted microscope system (Olympus, IX73).

A total of 3 × 10^5^ cells was plated into the 24-well plates for 24 h at −20 °C and each well was covered by a glass coverslip. Cells were fixed in 4% paraformaldehyde for 24 h at room temperature and blocked in 10% BSA for 2 h at room temperature. Cells were labeled with anti-Notch1 antibodies (1:100, Proteintech Catalog number: 10062–2-AP) at 4 °C for 12 h. The following day, cells were stained for 1 h with cy3-labeled goat anti-rabbit IgG (1:100; KeyGEN BioTECH, Nanjing, China) at 37 °C and slides were washed three times for 5 min with PBS. Nuclei were stained with DAPI (KeyGEN BioTECH) for 5 min at 37 °C. *POFUT1* immunofluorescence was imaged using a fluorescence microscope (Zeiss (ZEISS) LSM800 confocal microscope).

### Statistical analysis

Statistical analysis was performed using SPSS 19.0 software (SPSS Inc., Chicago, IL, USA). The data were imaged using Graph Pad Prism 5 software and expressed as the mean ± SD. Differences between the groups were compared using a two-tailed Student’s *t*-test. The expression levels of *shPOFUT1* groups were independently calculated relative to those of NC groups, which were normalized to 1; bar, SD; *p < 0.05, **p < 0.03, ***p < 0.01. (*t*-test). *p* < 0.05 was considered statistically significant.
